# Which Moiety Drives Gangliosides to Form Nanodomains?

**DOI:** 10.1021/acs.jpclett.3c00761

**Published:** 2023-06-16

**Authors:** David Davidović, Mercedes Kukulka, Maria J. Sarmento, Ilya Mikhalyov, Natalia Gretskaya, Barbora Chmelová, Joana C. Ricardo, Martin Hof, Lukasz Cwiklik, Radek Šachl

**Affiliations:** † J. Heyrovský Institute of Physical Chemistry of the Czech Academy of Sciences, Dolejškova 2155/3, 182 00 Prague, Czech Republic; ‡ Faculty of Science, Charles University, Hlavova 8, 128 40 Prague, Czech Republic; § Faculty of Chemistry, Jagiellonian University, Gronostajowa 2, 30-387 Krakow, Poland; ∥ Instituto de Medicina Molecular, Faculdade de Medicina, Universidade de Lisboa, 1649-028 Lisbon, Portugal; ⊥ Shemyakin-Ovchinnikov Institute of Bioorganic Chemistry of the Russian Academy of Science, Miklukho-Maklaya 16/10, 117997 Moscow, Russia; # Faculty of Mathematics and Physics, Charles University, Ke Karlovu, 2027/3, 121 16 Prague, Czech Republic

## Abstract

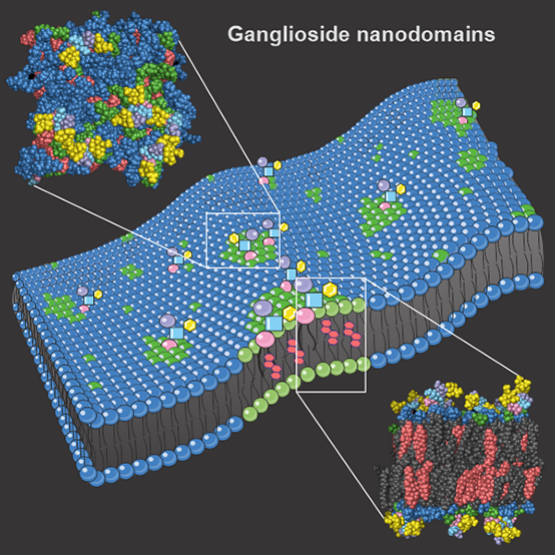

Gangliosides are important glycosphingolipids involved in a multitude of physiological functions. From a physicochemical standpoint, this is related to their ability to self-organize into nanoscopic domains, even at molar concentrations of one per 1000 lipid molecules. Despite recent experimental and theoretical efforts suggesting that a hydrogen bonding network is crucial for nanodomain stability, the specific ganglioside moiety decisive for the development of these nanodomains has not yet been identified. Here, we combine an experimental technique achieving nanometer resolution (Förster resonance energy transfer analyzed by Monte Carlo simulations) with atomistic molecular dynamic simulations to demonstrate that the sialic acid (Sia) residue(s) at the oligosaccharide headgroup dominates the hydrogen bonding network between gangliosides, driving the formation of nanodomains even in the absence of cholesterol or sphingomyelin. Consequently, the clustering pattern of asialoGM_1_, a Sia-depleted glycosphingolipid bearing three glyco moieties, is more similar to that of structurally distant sphingomyelin than that of the closely related gangliosides GM_1_ and GD_1a_ with one and two Sia groups, respectively.

Gangliosides are glycosphingolipids (GSLs) composed of a ceramide backbone and a bulky glycan headgroup containing at least one sialic acid (Sia) residue.^1−3^ They are abundant in neuronal plasma membranes, comprising ≤10–12% of all lipid species,^4^ and their biological function depends essentially on their ability to interact with soluble or membrane-associated molecules within the extracellular space.^2,5^ As a result, gangliosides are involved in a myriad of cellular processes, from adhesion, signaling, differentiation, and proliferation to Ca^2+^ homeostasis and cell-to-cell communication.^5−7^ Disruption of the correct presentation of gangliosides and the consequent impairment of their physiological interactions were already associated with multiple human pathologies, e.g., epilepsy, Alzheimer’s disease, Parkinson’s disease, and multiple sclerosis.^8−10^ Meanwhile, the literature suggests that the molecular presentation of gangliosides is controlled by their natural propensity to segregate laterally into membrane nanodomains.^11−16^ Moreover, within these nanodomains, gangliosides have been shown to interact with not only each other but also other sphingolipids, cholesterol, and transmembrane proteins.^1,17−20^ As expected, the polysaccharide group has been proposed as the primary catalyst for the nanoscopic segregation of gangliosides. It contributes to the energetic stabilization of the nanodomains by 65–67%, with the hydrogen bonding network formed at the headgroup level being considered its main stabilizer.^21^ However, published results are occasionally counterintuitive and even inconsistent with each other.^22−32^ The complexity of the matter is only underscored by the recent discovery that changes in the hydrophobic ceramide part of the molecule can also induce changes in the organization of gangliosides into nanodomains.^33^ It is thus not surprising that even today, the headgroups’ molecular moiety playing a crucial role in the formation of ganglioside nanodomains has not yet been identified. This severely limits our understanding of how to induce spontaneous nanoscopic lipid segregation, which occurs naturally in plasma membranes.^34,35^

In this study, we investigate the nanoscopic arrangement of gangliosides that share the same saccharide core chain and differ only in the number of Sia residues in the headgroup. To achieve high spatial resolution and molecular detail, we combine Förster resonance energy transfer analyzed by Monte Carlo simulations (MC-FRET)^36−40^ in giant unilamellar vesicles (GUVs) with atomistic molecular dynamics (MD) simulations. Thus, we identified the sialic acid moiety as a key group for the self-organization of gangliosides into nanodomains, significantly advancing our understanding of how the headgroup structure can enhance the propensity of gangliosides to segregate.

We started the study by examining the molecular structure of the most widely known ganglioside, GM_1_ (Figure 1). One molecular component that stands out in this respect is the Sia residue. In fact, the presence of this component in the molecular structure is a prerequisite for the molecule to be classified as a ganglioside. Our further considerations were guided by the experimental observation made on DPPC bilayers that the extent of domain formation increases with the number of Sia residues in the headgroup: GT_1b_ > GD_1a_ > GM_1_.^41−43^ Furthermore, because Sia moieties are potent hydrogen bond donors and acceptors, they are likely to contribute to the stability of the hydrogen bonding network formed between individual gangliosides.^21,29,44,45^ Thus, we designed this study so that we can observe the clustering and aggregation of gangliosides by investigating the nanoscopic arrangement of five different GSLs (asialoGM_1_ devoid of Sia, followed by GM_1_, GD_1a_, GT_1b_, and GQ_1b_ with one to four Sia residues, respectively) in synthetic bilayers with gradually decreased complexity. We focused primarily on the nanoscopic organization of gangliosides in quaternary DOPC/Chol/SM/GSLs (65/25/10/5 molar ratio) membranes containing excess bulk lipid DOPC and physiologically relevant amounts of cholesterol (Chol) and sphingomyelin (SM),^46−50^ selected for their pivotal roles in membrane function and biology, as well as one of the five studied gangliosides (Figure 1). To detect and characterize these nanoscopic structures in free-standing membranes of GUVs, we used MC-FRET (for more details, see the Supporting Information or refs (24) and (36−40)). With this approach, we estimate the size (radius ⟨*R*⟩) and membrane surface coverage (area fraction occupied by nanodomains ⟨*A*⟩) of the nanodomains by analyzing FRET between Bodipy-FL-C5 and Bodipy-564/570-C5 headgroup-labeled gangliosides (see Figure SI1 for the chemical structures). In keeping with our earlier research,^21^ we carried out the MC-FRET analysis independently for each GUV rather than averaging the data from all of the vesicles.

**Figure 1:**
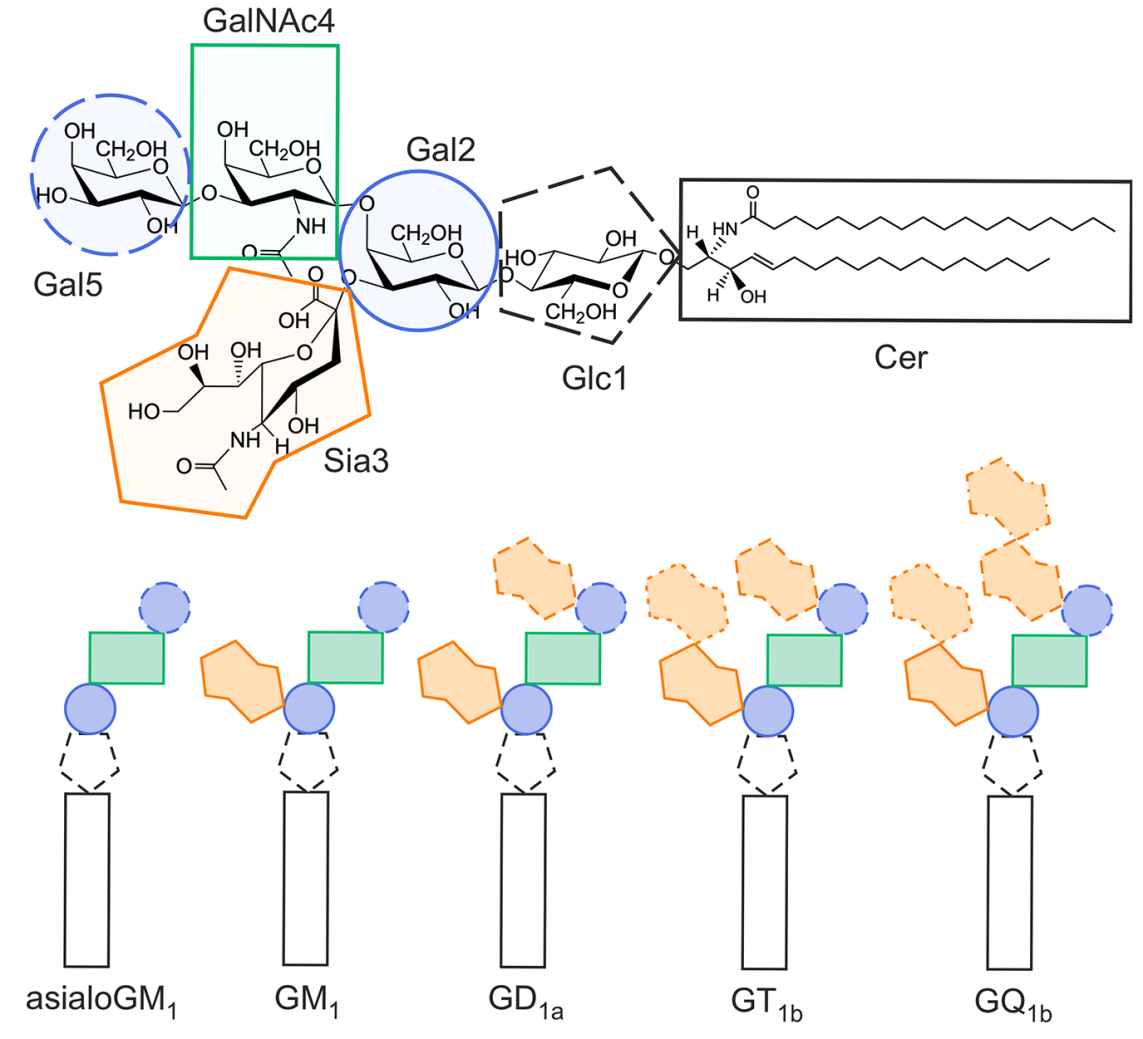
Cartoon representation of the studied gangliosides. Detailed structure of ganglioside GM_1_ (top), consisting of galactose (Gal), *N*-acetylgalactosamine (GalNAc), glucose (Glc), sialic acid (Sia), and ceramide (Cer). Five studied gangliosides (bottom) have Cer as the lipid backbone and differ in the number of Sia residues, from zero (asialoGM_1_, left) to four (GQ_1b_, right).

Figure 2 (left panels) shows that each of the five examined GSLs forms nanodomains ranging from 91 to 122 nm in radius ⟨*R*⟩, with an average surface area ⟨*A*⟩ of 45–54%. It implies that the chosen lipid composition might be too complex to enable the detection of differences in the behavior of distinct GSL species, and indeed, we identified sphingomyelin as a lipid that may contribute significantly to counteract the behavioral differences between GSL nanodomains, mainly because of its high concentration inside nanodomains but also because of its preferential interactions with gangliosides (see the Composition of ganglioside nanodomains section of the Supporting Information). Therefore, in the next step, we decided to simplify the membrane composition by replacing SM with additional DOPC (75/25/5 DOPC/Chol/GSL).

**Figure 2:**
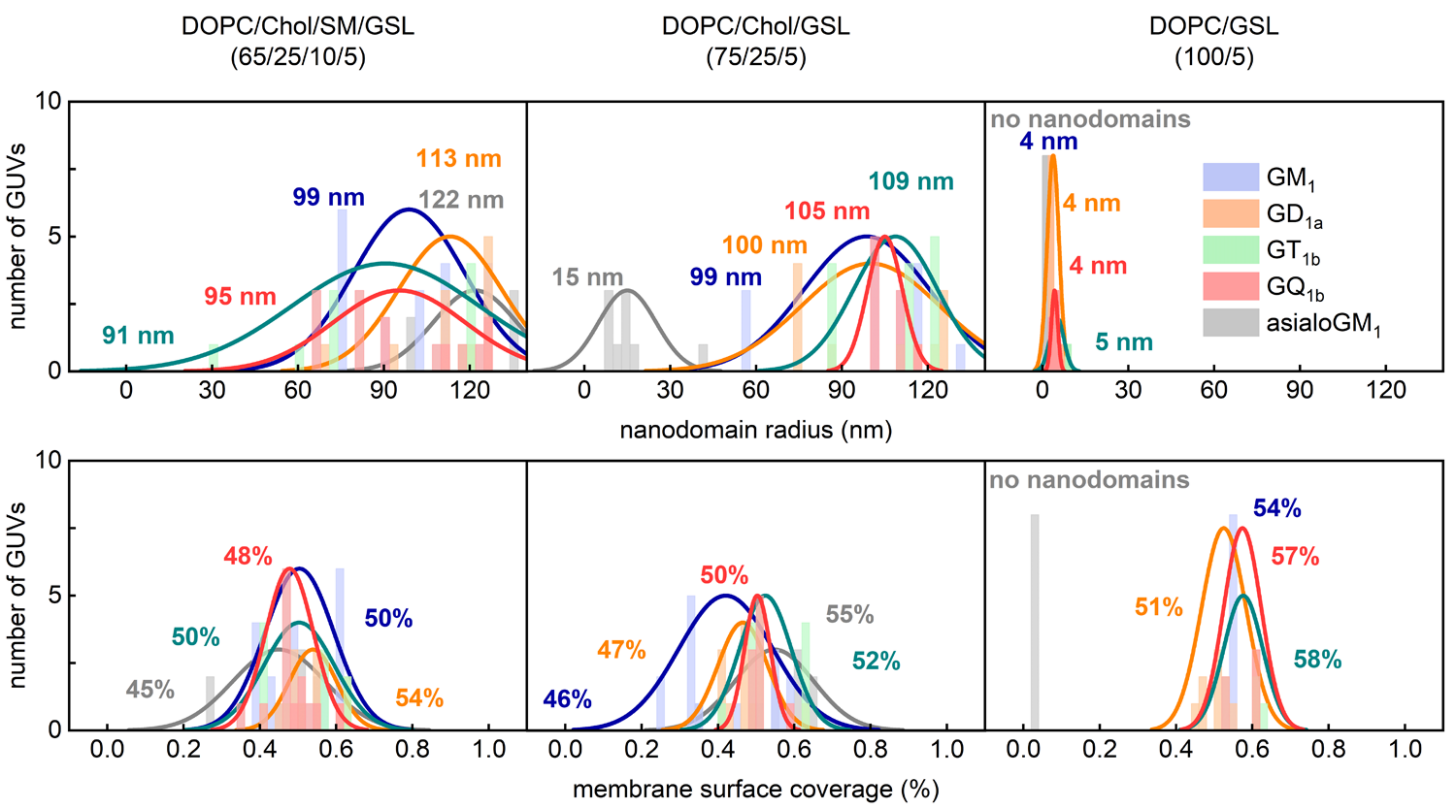
Frequency distribution plots of nanodomain size (top) and respective surface area coverage (bottom) for the five studied GSLs in GUVs of different composition. The bilayers contained 4 mol % asialoGM_1_ (gray), GM_1_ (blue), GD_1a_ (orange), GT_1b_ (green), or GQ_1b_ (red) and an additional 1 mol % of the GM_1_ FRET pair (0.5 mol % Bodipy-FL-C5 and 0.5 mol % Bodipy-564/570-C5 conjugated GM_1_). Every imaged GUV was analyzed by MC-FRET. Depending on variability, a total of 5–10 GUVs per lipid composition were imaged. The inset values represent the average nanodomain radius and the average surface coverage of the corresponding distributions.

In this case (Figure 2, central panels), both the nanodomain radius and the membrane surface coverage for all gangliosides except asialoGM_1_ mainly remained unaltered compared to those for the DOPC/Chol/SM/GSL membranes. In contrast, asialoGM_1_ was found to be sensitive to the absence of SM, showing a significant reduction in the radius of the nanodomains from 122 nm to only 15 nm, accompanied by a slight increase in the average surface area occupied by the domains (from 45% to 55%). Consequently, the first difference between GSLs with at least one Sia residue and asialoGM_1_ (without Sia) becomes apparent. In the context of these results, asialoGM_1_ seems to bear a striking resemblance to sphingomyelin, which in DOPC/Chol/SM (70/25/5) membranes organizes into nanodomains with an average radius of 9 nm occupying 45% of the membrane area.^38^ Similar features are also exhibited by nanodomains formed by ganglioside GM_3_ (for the structure, see Figure SI5), which, although it contains one Sia moiety, has a greatly reduced sugar head containing only Glc-Gal2-Sia3 sugar residues.^21^

Then, we set out to check whether the observed similarities between asialoGM_1_ and SM would hold under different conditions. In an attempt to further destabilize the nanodomains, we removed all cholesterol from the membrane, taking the experiment to an extreme because the membrane simplified in this way ultimately contained only the bulk lipid DOPC and the given ganglioside (100/5 DOPC/GSL). The MC-FRET analysis (Figure 2, right panels) revealed that all Sia-containing GSLs formed nanodomains in binary DOPC/GSL bilayers, with a restricted range of average radius around 5 nm, whereas no nanodomains were observed for asialoGM_1_ (⟨*A*⟩ close to 0), which is in line with the clustering behavior of SM.^38^ Moreover, in simple DOPC/GSL membranes, the similarity between asialoGM_1_ and ganglioside GM_3_ is lost, as GM_3_ does not cease to organize into nanoscopic domains (⟨*R*⟩ = 19 nm, and ⟨*A*⟩ = 61%) even in pure DOPC vesicles.^21^ Considering the fact that asialoGM_1_ still contains four sugars in the glycan headgroup, it is surprising that its clustering pattern is more similar to that of SM, in which the headgroup is completely replaced by phosphatidylcholine (PC), than to that of the closely related ganglioside GM_1_ or the structurally similar GM_2_ and GM_3_.

To understand the reasons for this different clustering behavior of asialoGM_1_ compared to that of other GSLs, we probed the organization of the clusters employing atomistic MD simulations. As our previous study showed that the stability of the ganglioside nanodomains is strongly associated with the ability of GSLs to form intermolecular interactions, here we focused primarily on the analysis of hydrogen bonds between individual gangliosides.^21^ MD was carried out for asialoGM_1_, GM_1_, and GD_1a_ in DOPC/Chol/SM/GSL (65/25/10/5) membranes; however, we took into account only the bilayer closely resembling the interior of the ganglioside nanodomains because the full size of nanodomains is too vast to be reproduced in all-atom simulations. See the Supporting Information for a complete set of data and computational details.

The calculated average number of hydrogen bonds between GSL molecules shows that the total number of interactions increases with the number of Sia moieties (Table 1). A detailed analysis of the results presented in Table 1 revealed that the Sia moiety is indeed responsible for the highest number of molecular interactions formed in all studied systems, i.e., two hydrogen bonds per Sia residue. In contrast, the number of hydrogen bonds formed by Glc1 and Gal2 groups ranges between 0.4 and 0.7 in most cases. It is worth noting that the GalNAc4 moiety is also crucial for altering the number of hydrogen bonds formed between GSL molecules. Comparison between GM_1_ and GM_3_ (with and without GalNAc4, respectively) demonstrates that in the presence of GalNAc4, the number of interactions is reduced from 1.4 to 0.4 on Gal2 and from 2.6 to 2.1 on Sia3 (Table 1). In this respect, asialoGM_1_ is thus at a disadvantage regarding the total number of hydrogen bonds, because it not only lacks the Sia moiety that strongly mediates the hydrogen bonding network but also contains a GalNAc4 group that inhibits the formation of hydrogen bonds between neighboring groups. Hence, compared to the structurally related GM_1_, asialoGM_1_ forms considerably fewer hydrogen bonds with nearby GSLs (2.8 vs 6.2) and, despite containing four sugar groups, asialoGM_1_ forms significantly fewer hydrogen bonds (2.8) than does GM_3_ (4.7), whose glycan headgroup consists of only three sugar moieties. Paradoxically, asialoGM_1_, mediating 2.8 hydrogen bonds on average, is more similar in its propensity to form hydrogen bonds to the structurally more distant SM, which mediates only one hydrogen bond, than to the closely related GM_1_, with 6.2 bonds. To support the results presented above, we quantified the interaction energy between individual GSLs differing in the number of Sia groups in their structure (Table 2 and Figure SI3). The presence of Sia considerably increases the final interaction energy (more negative). This trend is evident despite a significant scatter in the data caused by the heterogeneous population of ganglioside clusters present in MD simulations (Figure SI3). Specifically, the attachment of the Sia moiety to asialoGM_1_ (which gives rise to GM_1_) leads to an ∼60% increase in the interaction energy, even though Sia represents only one of the five sugar moieties in the headgroup. This effect, however, is not additive because in the case of GD_1a_, which contains six sugar units, the addition of two Sia residues increases the interaction energy considerably but on average only ∼46% (Table 2 and Figure SI3).

**Table 1:** Average Numbers of Hydrogen Bonds per GSL Sugar Moiety for AsialoGM_1_, GM_3_, GM_1_, and GD_1a_ in DOPC/Chol/SM Bilayers^a^

GSL	Sia6	Gal5	GalNAc4	Sia3	Gal2	Glc1	total	SM–SM
GD_1a_	2.5 ± 0.0	1.6 ± 0.0	1.1 ± 0.0	2.2 ± 0.1	2.1 ± 0.0	0.5 ± 0.0	10.0	1.0
GM_1_	–	2.0 ± 0.1	1.1 ± 0.0	2.1 ± 0.1	0.4 ± 0.0	0.6 ± 0.0	6.2	1.0
GM_3_^b^	–	–	–	2.6 ± 0.2	1.4 ± 0.1	0.7 ± 0.1	4.7	–
asialoGM_1_	–	1.0 ± 0.0	0.9 ± 0.0	–	0.4 ± 0.0	0.5 ± 0.0	2.8	1.0

a The corresponding results for the DOPC and DOPC/Chol membranes are almost identical (see Table S2).

b Data for GM_3_ adopted from ref (21).

**Table 2:** Average Interaction Energies (kilojoules per mole) between the GSL Sugar Headgroups (marked as homogeneous) and between the GSL Sugar Headgroups and the Surrounding Bulk Lipids (heterogeneous) in the Simulated Membranes^a^

	homogeneous interactions		heterogeneous interactions					
GSL	GSL/GSL	GSL/GSL_norm_	DOPC	DOPC_norm_	SM	SM_norm_	Chol	Chol_norm_
GD_1a_	–51	–4.3	–298	–3.3	–86	–3.3	–78	–1.2
GM_1_	–56	–4.7	–247	–2.7	–123	–4.7	–83	–1.3
asialoGM_1_	–35	–2.9	–267	–3.0	–101	–3.9	–88	–1.4

a The total energy per GSL and the value normalized by the number of interacting partners (subscript norm) are given. The spread of the GSL–GSL interaction energy values is quantified in Figure SI3.

As part of this study, we also computed standardized pairwise interaction energies per lipid to allow for the comparison of pairwise interaction energies for homogeneous GSL/GSL and heterogeneous GSL/bulk DOPC, Chol, or SM pairs (Table 2). Again, asialoGM_1_ stands out from this comparison. In fact, the interaction energy values for asialoGM_1_/asialoGM_1_ are similar to those of asialoGM_1_/DOPC or asialoGM_1_/SM, contradicting the exclusivity of homogeneous interactions for the stability of GSL nanodomains. This contrasts with the interaction energies for GM_1_/GM_1_ or GD_1a_/GD_1a_; the interaction energy of each is significantly more negative than the interaction energies for the heterogeneous GM_1_(GD_1a_)/DOPC pairs.

Altogether, these findings imply that the potential of gangliosides to organize into nanoscopic domains coincides very closely with the improved ability of the Sia moiety to form hydrogen bonds. Consequently, asialoGM_1_ has a major disadvantage compared to gangliosides that contain at least one Sia group, in terms of both the number of hydrogen bonds stabilizing the GSL nanodomains and the interaction energy, which is significantly lower for asialoGM_1_ than for the other studied gangliosides.

As gangliosides in general^51^ and specifically the Sia moiety^8^ act as receptors for membrane binding proteins, we were further interested in how the molecular structure of the investigated ganglioside influences their ability to form hydrogen bonds with the surrounding bulk water. Specifically, we determined the number of hydrogen bonds among water, Sia groups, and the entire sugar headgroup (Table 3). As follows from Table 3, the average number of all hydrogen bonds formed between Sia groups and water (per GSL) in the nanodomains is ∼12, which is slightly more than 50% of all hydrogen bonds formed by the entire “core” group (headgroup without Sia). AsialoGM_1_, which lacks the Sia group, forms 33% fewer contacts with water than the closely related GM_1_, which contains one Sia group. Compared with GD_1a_, which contains two Sia residues, asialoGM_1_ presents only half of the number of contacts with water. All of these values are in strong contrast to the number of hydrogen bonds between the SM headgroup and water (only 4.5). The sialic group thus mediates contacts with the environment in a fundamental way and contributes significantly to not only the stability of ganglioside nanodomains but also the formation of contacts with the surrounding water molecules.

**Table 3:** Average Numbers of Hydrogen Bonds between Sia and Water and the Corresponding Values for Whole Headgroup–Water Hydrogen Bonds per GSL Molecule^a^

		GSLs in nanodomains		one isolated GSL molecule	
GSL		no. of hydrogen bonds	fraction formed by Sia (%)	no. of hydrogen bonds	fraction formed by Sia (%)
GD_1a_	headgroup	43.5 ± 0.4	–	44.6 ± 0.4	–
	Sia1	12.1 ± 0.1	27.8	12.6 ± 0.2	28.3
	Sia2	12.6 ± 0.0	29.0	13.5 ± 0.2	30.3
GM_1_	headgroup	32.6 ± 0.1	–	34.3 ± 0.2	–
	Sia1	12.2 ± 0.0	37.4	13.1 ± 0.1	38.1
asialoGM_1_	headgroup	22.0 ± 0.4	–	22.9 ± 0.7	–
SM	headgroup	4.3 ± 0.0	–	4.5 ± 0.0	–

a The percentage of hydrogen bonds that are established by the sialic group relative to the total number of bonds formed by the headgroup is also presented. The analysis was carried out on the GSLs contained inside the nanodomains as well as on an isolated GSL molecule found in the DOPC/Chol/SM (65/25/10) bilayer.

In the final part of this study, we set out to determine the extent to which the interactions of GSLs with the surroundings described above are influenced by the formation of the nanodomains. For example, ganglioside-mediated host–pathogen interactions^10,52^ depend on the precise identification of ganglioside receptors by ganglioside binding motifs. More specifically, the dendritic cell protein Siglec-1 (sialic acid binding Ig-like lectin 1) recognizes gangliosides on the viral membrane of enveloped viruses like the human immunodeficiency virus (HIV-1) or the Ebola virus,^53^ assisting in the propagation of viral infection and the antiviral immune response. Another well-known example is the interaction of the cholera toxin protein with five GM_1_ molecules when it approaches cellular plasma membranes. In this specific case, it has already been established that the membrane composition and organization influence these interactions.^6^ Therefore, we extended the aforementioned analysis of hydrogen bonds formed by gangliosides within nanodomains (left part of Table 3) to the analysis of hydrogen bonds for an isolated ganglioside molecule located in a lipid layer of the same lipid composition (right part of Table 3). In this way, we intended to test the extent to which nanodomains influence the accessibility of the ganglioside by ligands approaching from the bulk solution. Interestingly, after the isolation of one GSL molecule in the lipid bilayer, the number of contacts between the headgroup and water remains almost unchanged (Table 3). We complemented this investigation by comparing calculated normalized partial density profiles of specific ganglioside moieties for both the GSL present in the nanodomains and the isolated GSL molecule (for more details, see Figure SI4). These results suggest that although there is a stretching of the terminal groups of gangliosides in the nanodomains, this does not result in better accessibility of the glycan group to the surrounding water. Thus, the conclusion we reached in the previous study^21^ for gangliosides GM_1_–GM_3_, namely that the biological role of ganglioside nanodomains is not to expose the Sia more efficiently to the solvent but rather to provide a nanoscopic platform with an increased local concentration of ganglioside receptors, can be generalized to the gangliosides studied here.

Overall, these results show that the sialic acid group, which is an integral part of the bulky sugar headgroup of the ganglioside molecule, is crucially involved in the formation of ganglioside nanodomains. This conclusion can be unambiguously drawn by both experiments and simulations. The MC-FRET experiments show that the clustering pattern of asialoGM_1_, which lacks any Sia group, in lipid bilayers with ternary and binary lipid composition is fundamentally different from those of all of the other examined gangliosides (GM_1_–GM_3_, GT_1b_, GD_1a_, and GQ_1b_) and is similar, in many respects, to that of the distantly related sphingomyelin, in which the glycan head is completely replaced by a PC headgroup. For example, neither asialoGM_1_ nor SM forms nanodomains in a homogeneous DOPC bilayer.^38^ This observation sharply contrasts with those for GM_1_–GM_3_, GT_1b_, GD_1a_, and GQ_1b_, which readily segregate into <10 nm domains. MD simulations showed that gangliosides interact with each other through a complex net of hydrogen bonds formed side by side, with the Sia moiety being responsible for most of these molecular interactions. AsialoGM_1_ not only lacks the Sia group, a potent mediator of hydrogen bonds, but also contains a GalNAc4 group, which inhibits the formation of hydrogen bonds on neighboring sugar moieties. Consequently, asialoGM_1_’s capacity to generate hydrogen bonds (∼2.8 bonds on average) further resembles that of the structurally more distant SM (1 hydrogen bond) than it does that of the closely related GM_1_, with ∼6.2 bonds. Furthermore, this singularity is even more evident when comparing the number of hydrogen bonds of asialoGM_1_ and GM_3_. Strikingly, with only three sugar groups, GM_3_ forms an average of ∼4.7 hydrogen bonds, while asialoGM_1_ containing four sugars forms only ∼2.8 bonds.

MD simulations further show that the Sia residue also functions as the key mediator of interactions of the ganglioside with its surroundings. GD_1a_, which contains two Sia groups in its structure, binds twice as many water molecules as asialoGM_1_. There is also a significant difference between GM_1_ and asialoGM_1_. AsialoGM_1_ forms ∼30% fewer pairs with water than does GM_1_. The formation of nanodomains leads to only modest changes in the glycan headgroup presentation, and the number of interactions between the glycan headgroup and the surrounding water is still conserved. Thus, it seems likely that the loss of hydrogen bonds between water and the glycan head due to the arrangement of GSL in the nanodomains is effectively compensated by the exposure of the ganglioside terminal groups to the aqueous environment. The resulting nanodomains, therefore, do not appear to function as units that improve the access to functional ganglioside groups but rather as interaction platforms that localize gangliosides to specific sites.

## Experimental Section

The material used in the study is summarized in the Supporting Information, including experimental details on MC-FRET measurements and analysis as well as details of MD simulations.

## Supporting Information Available

Experimental details of sample preparation, MC-FRET measurements and quantitative analysis of time-resolved fluorescence decays, details of MD simulations, and additional results supporting the statements made in the text (PDF)
Transparent Peer Review report available (PDF)
