# Supplementary material for: Which Moiety Drives Gangliosides to Form Nanodomains?
Source: J Phys Chem Lett. 2023 Jun 16;14(25):5791–7. doi: 10.1021/acs.jpclett.3c00761 (PMC10316399; doi:10.1021/acs.jpclett.3c00761)
Supplement: Supplementary file 1 — jz3c00761_si_001.pdf [file jz3c00761_si_001.pdf]

Supporting Information

Which Moiety Drives Gangliosides to Form Nanodomains?

David Davidović[1,2,#], Mercedes Kukulka[3#], Maria J. Sarmento[4#], Ilya Mikhalyov[5], Natalia Gretskaya[5], Barbora Chmelová[1,6], Joana C. Ricardo[1], Martin Hof[1], Lukasz Cwiklik[1,*] and Radek Šachl[1,*]

[1] J. Heyrovský Institute of Physical Chemistry of the Czech Academy of Sciences, Dolejškova 2155/3, 182 00 Prague, Czech Republic

[2] Faculty of Science, Charles University, Hlavova 8, 128 40 Prague, Czech Republic

[3] Faculty of Chemistry, Jagiellonian University, Gronostajowa 2, 30-387 Krakow, Poland

[4] Instituto de Medicina Molecular, Faculdade de Medicina, Universidade de Lisboa, 1649-028 Lisbon, Portugal.

[5] Shemyakin-Ovchinnikov Institute of Bioorganic Chemistry of the Russian Academy of Science, Miklukho-Maklaya 16/10, 117997 Moscow, Russia

[6] Faculty of Mathematics and Physics, Charles University, Ke Karlovu, 2027/3, 121 16 Prague, Czech Republic

[#]contributed equally

***Correspondence**

Radek Šachl: radek.sachl@jh-inst.cas.cz

Lukasz Cwiklik: lukasz.cwiklik@jh-inst.cas.cz

Table of contents

Experimental section 2

GUV preparation and immobilization 2

Fluorescence lifetime imaging microscopy measurements 3

Characterization of nanodomains by MC-FRET 3

MD simulations 4

Materials 5

Supporting Results 6

The composition of ganglioside nanodomains 6

The interaction energies between GSL sugar headgroups 8

The average number of hydrogen bonds in DOPC/Chol and DOPC bilayers 8

The analysis of the tilt angle of the sialic acid vector from the bilayer normal 8

Supporting Figures 10

References 10

Experimental section

GUV preparation and immobilization

Lipid stock solutions were prepared in chloroform, apart from all labelled and unlabelled gangliosides that were prepared in chloroform/methanol 2:1 (v/v). Giant unilamellar vesicles (GUVs) were obtained by electroformation as previously described [1]. Briefly, lipid mixtures were prepared in chloroform from lipid stock solutions (100 nmol in ~100 µL) and spread on two hollow titanium plates. To facilitate solvent evaporation, the plates were placed on a heating plate at ~35 °C and then left in vacuum for at least 1h to assure the evaporation of any remaining traces of solvents. Afterwards, the lipid-coated plates were assembled using parafilm as insulator. This electroswelling chamber was then filled with 1 mL of sucrose solution (103 mOsm/kg) and sealed with parafilm. For GUV electroformation, an alternating 10 Hz electrical field rising from 0.02 V to 1.1 V (peak-to-peak) was first applied during the first 45 min, and then kept at 1.1 V for 1.5h. The subsequent detachment phase was set at 4 Hz and 1.3 V for 30 min. The entire electroformation procedure was carried out at 47 °C. Upon formation, GUVs were transferred to an 8-well µ-Slide from Ibidi (Munich, Germany) containing glucose buffer (~80 mM glucose, 10 mM HEPES and 10 mM NaCl, pH 7.2) at 103 mOsm/kg. In order to immobilize the vesicles, the slide chambers were previously coated with BSA-biotin/streptavidin and 0.5 mol% of DOPE-biotin was included in all lipid mixtures. For FLIM-FRET measurements, both donor and acceptor fluorescently labelled lipids were used at 1:200 of total lipid, if not stated otherwise.

Fluorescence lifetime imaging microscopy measurements

Fluorescence lifetime imaging microscopy (FLIM) measurements were carried out on a home-built setup consisting of an inverted confocal microscope body IX71 (Olympus, Hamburg, Germany) and a pulsed diode laser (LDH-P-C-470, 470 nm, PicoQuant, Berlin, Germany) operated at a 10 MHz repetition rate. Laser intensity was 2 µW to avoid any pile-up effects. The laser light was coupled to a polarization-maintaining single-mode optical fiber and re-collimated at the output with an air space objective (UPLSAPO 4x, Olympus). The light was up reflected onto a water immersion objective (UPLSAPO 60x, Olympus) with a 470/635 dichroic mirror. The donor emission signal was selected using a 515/50 bandpass filter (Chroma Rockingham, VT) and detected by a single photon avalanche diode. For each GUV, a 512x512 pixels image was acquired (0.6 ms/pixel) at the vesicle's cross-section. An experimental donor fluorescence decay was also obtained. A minimum of 5 vesicles were measured per composition. All measurements were performed at 25 °C.

Characterization of nanodomains by MC-FRET

The whole analysis is described in detail in [2] and is based on both the use of fluorescently labeled gangliosides serving as donors (Bodipy-FL-GM_1) and acceptors (Bodipy-564/570-GM_1) in a FRET pair and the fact that formation of ganglioside nanodomains will lead to the accumulation of donors and acceptors within the nanodomains. This will bring the donors closer to the acceptors and enhance the efficiency of FRET. The formation of nanodomains thus influences the kinetics of fluorescence deexcitation of the donors if they are near the acceptors. According to our previous work, the shape of the donor decay contains information about the average nanodomain size, the total area occupied by the nanodomains, and their inter-leaflet organization. With FRET a 'yes or no' answer regarding the presence of nanodomains can be obtained by a simple comparison of fluorescence decays or average fluorescence intensities of donors in the presence and absence of acceptors. However, to obtain information about the size, concentration, and interleaflet coupling of the nanodomains, time-resolved fluorescence decays must be recorded and analyzed by Monte Carlo simulations. This approach is then referred to as MC-FRET [2-6]. The following assumptions are made during the MC-FRET analysis: first, nanodomains are assumed to be uniform in size. Although this extreme assumption appears unlikely, using a fitting model that considers a distribution of nanodomain sizes does not result in better fit outcomes. Second, the nanodomains are assumed to be circular. Such circular nanodomains are considered basic building blocks, the assembly of which might form larger, non-circular-shaped structures that correspond better to a real situation.

MD simulations

All-atom MD simulations, as well as the subsequent analyses, were performed using the GROMACS 2021.2 program package [7]. The bilayers containing the GD_{1a}, GM_1 and asialoGM_1 gangliosides at two concentrations and three membrane compositions (DOPC/SM/Chol, DOPC/Chol and DOPC) were set up using the CHARMM-GUI builder [8-10]. The exact number of lipid molecules in each system is shown in Table S1. Each bilayer was solvated with 14,000 water molecules. Na^+ and Cl^- ions were added to neutralize the net charge and represent the physiological salt concentration of 150 mM. The CHARMM36m force field parameters [11] were used for ganglioside molecules, DOPC, SM and Chol, whereas the TIP3P model was applied for water [12].

Prior to the MD simulations, the energy minimization for all systems was performed over 5000 steps using the steepest-descent method, followed by the initial equilibration of temperature conducted under the NVT ensemble with the Berendsen thermostat for 250 ps, and subsequently pressure under the NPT ensemble with the pressure coupling of Berendsen for 625 ps.

MD simulations were performed using the leap-frog algorithm with a time step of 2 fs for 2.5 and 0.1 µs for the concentrated and diluted mode, respectively. All hydrogen-involving bonds were constrained using the LINCS algorithm [13]. The Nose-Hoover thermostat [14, 15] was combined with the Parrinello-Rahman barostat [16] to keep the temperature constant at 310 K and the pressure at 1 bar. The coupling time constants were set to 1.0 and 5.0 ps, respectively. Semi-isotropic pressure coupling in the bilayer plane and along the normal to the membrane was used. Long-range electrostatic interactions were computed using the Particle-Mesh Ewald summation [17] with a cut-off of 1.2 nm.

In each simulated trajectory, equilibration was monitored by means of intermolecular contacts and radial distribution functions. The following analyses of the obtained trajectories were carried out for the last 500 ns of each concentrated mode (and 50 ns of the diluted mode) simulation using GROMACS tools:

- gmx hbond for the number of hydrogen bonds
- gmx density for the partial density of each lipid moiety along the z-axis
- gmx rdf for the radial distribution of lipids around the reference atom in each lipid as follows: for DOPC the second carbon of the glycerol backbone, for GSL and SM the CH carbon of the sphingosine backbone, and for Chol the carbon attached to the OH group.
- gmx gangle for the cosine of the tilt angle of the ganglioside from the normal of the membrane. The tilt angles were defined as the angles between the amide nitrogen atoms of the Sia moieties, the O1 of Glc1 and the ray originating from O1 of Glc1 in the direction parallel to the z-axis.

Table S1. Molecular composition of the simulated systems expressed in the number of lipid molecules present in the simulation box.

	**DOPC**	**SM**	**Chol**	**GSL**	
				**diluted mode**	**concentrated mode**
DOPC/SM/Chol	90	26	64	2	12
DOPC/Chol	112	-	76	2	16
DOPC	118	-	-	2	10

Materials

1,2-dioleoyl-sn-glycero-3-phosphocholine (DOPC), GM_1 ganglioside (bovine brain sodium salt), N-stearoyl-D-erythro-sphingosylphosphorylcholine (SM), cholesterol (ovine wool) and 1,2- dipalmitoyl-sn-glycero-3-phosphoethanolamine-N-(cap biotinyl) (biotinyl-PE) were purchased from Avanti Polar Lipids (Alabaster, AL, USA). Biotin labelled bovine serum albumin (biotinyl-BSA) and other chemicals (HEPES, D-glucose, sucrose, NaCl, HCl) were purchased from Sigma Aldrich (St. Louis, USA). All other bovine brain gangliosides (asialoGM_1 semisynthetic from GM_1, GD_{1a} disodium salt, GT_{1b} trisodium salt and GQ_{1b} tetrasodium salt) were purchased from Enzo Life Sciences Inc. (Farmingdale, New York). Organic solvents of spectroscopic grade were purchased from Merck (Darmstadt, Germany). Streptavidin was purchased from Iba Lifesciences (Gottingen, Germany). All chemicals were used without further purification. Fluorescent probes BODIPY-FL-C5-GM_1 and BODIPY-564/570-C5-GM_1 (**Figure SI1**) were synthesized by Dr. Ilya Mikhalyov and Dr. Natalia Gretskaya as described in [18]. All solvents used here were of spectroscopic grade.

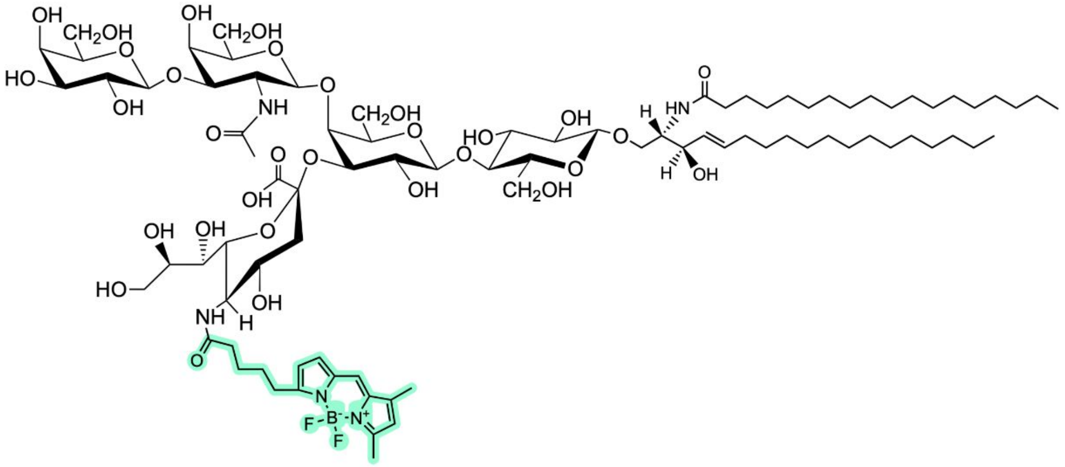

BODIPY-FL-C5-GM_1

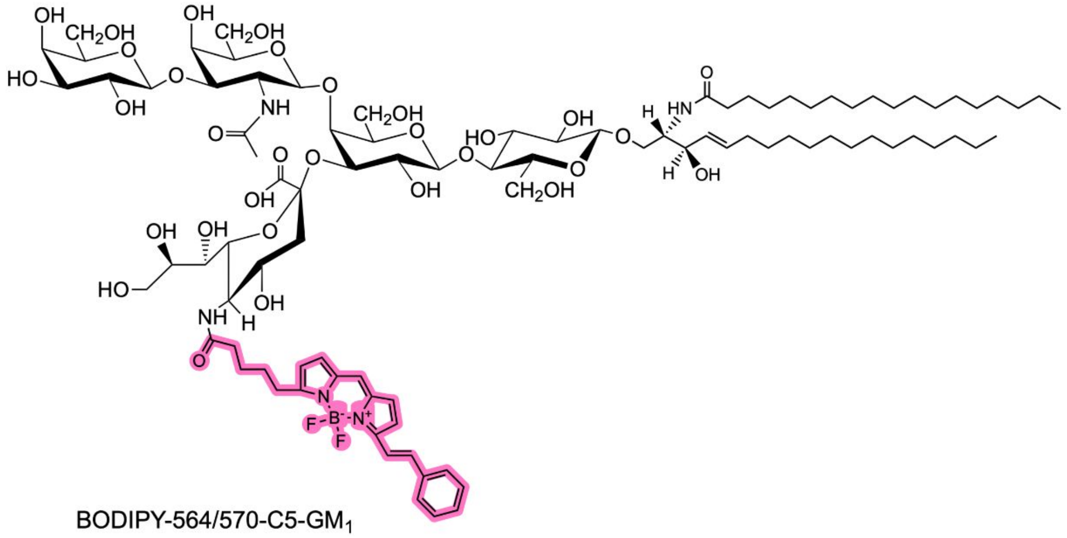

BODIPY-564/570-C5-GM_1

Figure SI1: Chemical structures of fluorescently labelled GM_1 gangliosides used in this work as donors (BODIPY-FL-C5-GM_1) and acceptors (BODIPY-564/570-C5-GM_1) of FRET.

Supporting Results

The composition of ganglioside nanodomains

Since direct quantification of individual molecules within nanodomains is beyond our experimental possibilities, we instead calculated in-membrane plane radial distribution functions $g(r)$ for the DOPC, Chol, and SM molecules surrounding GSLs based on the data obtained from MD simulations. These functions provide insight into the distribution of lipids around each ganglioside and, consequently, the composition of GSL nanodomains. The amplitude of $g(r)$ is proportional to the probability of finding a particle at a given distance from a reference particle. The $g(r)$ function is defined as having a value of 1 for an isotropic lipid distribution and a value greater than 1 for an accumulation of lipids.

As seen in **Figure SI2**, all $g(r)$ functions exhibit a peak at 0.25-0.50 nm, indicating that the nanodomains consist of all membrane components. The largest amplitude of the peak is observed for SM, implying preferential interactions between GSLs and SM, and thus accumulation of SM in ganglioside nanodomains. Chol, on the other hand, is able to approach the closest to GSLs of all membrane components, probably due to its small size, while DOPC appears to have neither preferential nor repulsive interactions with the gangliosides. Given that ganglioside nanodomains account for more than 50% of the lipid bilayer surface and that gangliosides make only up to 5% of all lipids in the membrane, the formed nanodomains must also contain a significant amount of DOPC lipids. Therefore, despite being called ganglioside nanodomains, these heterogeneities primarily consist of other lipid components, such as DOPC, SM, and Chol. However, as demonstrated in the main manuscript, gangliosides, particularly their bulky headgroup with one or more Sia acid residues, play a significant role in stabilizing these nanodomains.

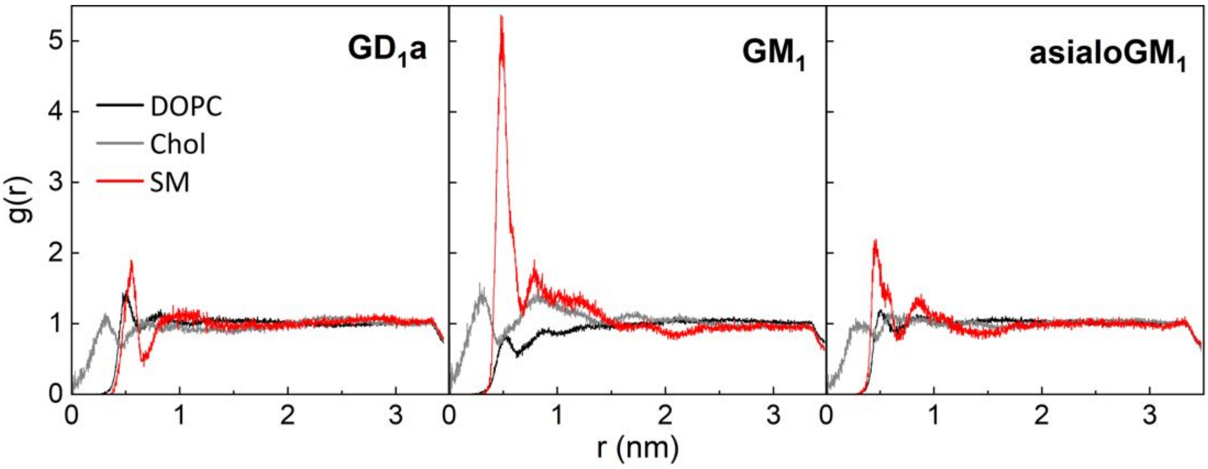

Figure SI2: In-membrane plane radial distribution functions, g(r), of DOPC, Chol, and SM around GSL in the DOPC/SM/Chol bilayer.

The interaction energies between GSL sugar headgroups

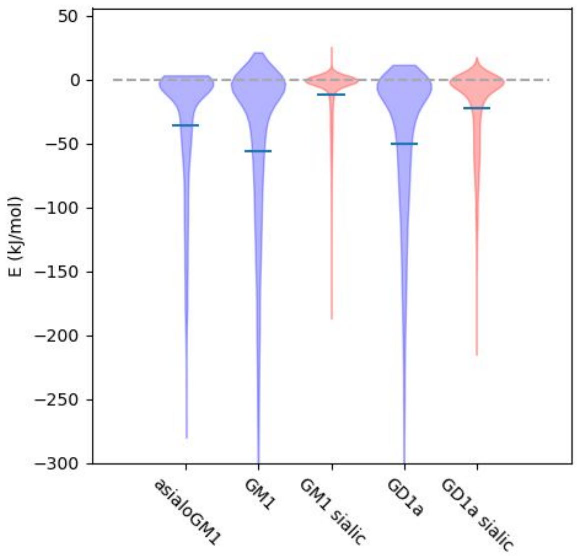

Figure SI3: Interaction energies between GSL sugar headgroups in the simulated membranes, presented using violin plots, thus quantifying the spread of the interaction energy values caused by the heterogeneous population of ganglioside clusters. The energy per one GSL headgroup is given. For GM1 and GD1a, also the interaction energy between their sialic moieties and other GSL headgroups is reported (in red). The dashed line marks the zero value, and the blue lines show the average values (as given in Table 2).

The average number of hydrogen bonds in DOPC/Chol and DOPC bilayers

Table SI2. Average number of hydrogen bonds per GSL sugar moiety for asialoGM$_1$, GM$_1$, and GD$_{1a}$ in DOPC/Chol and DOPC bilayers.

Bilayer	GSL	Sia6	Gal5	GalNAc4	Sia3	Gal2	Glc1	Total
DOPC/Chol	GD$_{1a}$	2.4 ± 0.0	1.6 ± 0.0	1.2 ± 0.0	2.2 ± 0.1	2.0 ± 0.1	0.5 ± 0.0	9.9
	GM$_1$	-	2.1 ± 0.1	1.1 ± 0.1	2.3 ± 0.2	0.5 ± 0.0	0.5 ± 0.0	6.5
	asialoGM$_1$	-	1.1 ± 0.0	1.0 ± 0.0	-	0.5 ± 0.0	0.5 ± 0.0	3.2
DOPC	GD$_{1a}$	2.2 ± 0.1	1.4 ± 0.1	1.2 ± 0.0	2.2 ± 0.1	2.0 ± 0.1	0.5 ± 0.0	9.2
	GM$_1$	-	2.2 ± 0.1	1.4 ± 0.0	2.3 ± 0.1	0.5 ± 0.0	0.7 ± 0.0	7.1
	asialoGM$_1$	-	1.1±0.0	0.9±0.0	-	0.4 ± 0.0	0.5 ± 0.0	3.0

The analysis of the tilt angle of the sialic acid vector from the bilayer normal

An analysis of the tilt angle of the sialic acid vector (defined from the O1 atom of glucose to the nitrogen atom of sialic acid) from the normal to the membrane surface supports the determined localization of the sialic acid. Namely, the MD simulations of GD$_{1a}$ (**Figure SI4**) revealed that the tilt of sialic acids becomes more deviated from the perpendicular orientation to the membrane surface ($\cos(\theta)=1$) in the isolated molecule in comparison with the GSL molecule located within the nanodomains. As a result, the arrangement of GSL molecules in nanodomains causes a modest stretching of the terminal parts of the glycan head, including the Sia group. Similarly to what our

previous study showed for the GM_1, GM_2 and GM_3 molecules [19], the relative positions of the peaks maxima from the centre (midplane) of the lipid bilayer for the terminal glycans (Gal5 and Sia6) are visibly shifted towards lower values for the isolated molecule compared to the GSL molecule located within the nanodomains. For the other moieties, the maxima remain almost the same. This indicates that the Gal5 and Sia6 residues are localized closer to the bilayer surface when the GLS molecule is isolated. In fact, the analysis of the tilt angle of the sialic acid vector showing a change from the normal to the membrane surface in the isolated molecule further supports this observation (**Figure SI4 C,D**)

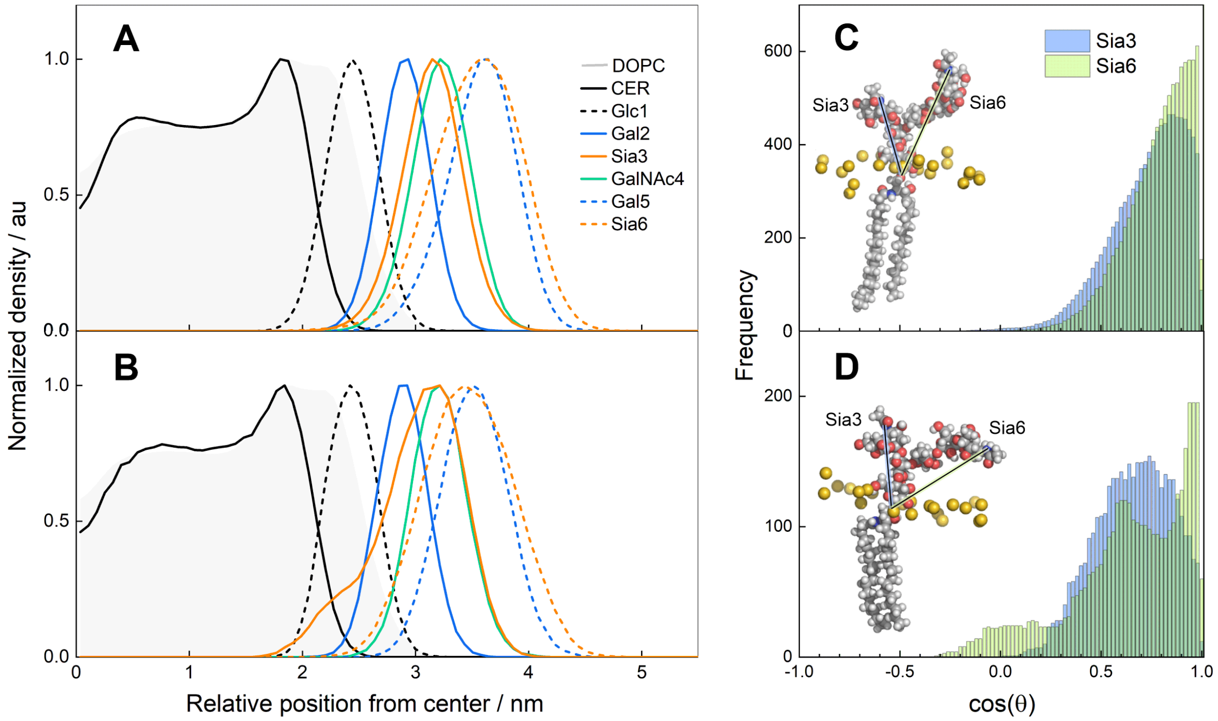

Figure SI4: Left: Normalized partial density profiles for DOPC and individual moieties of ganglioside molecules located in the nanodomains (A) and for an isolated ganglioside molecule (B), averaged for the top and bottom leaflets. Right: Histogram of cos(θ), describing the tilt angle of the sialic acids in relation to the normal of the membrane, both in the concentrated (C) and diluted (D) mode. The results are presented for GD1a in the DOPC/SM/Chol bilayer. Insets: Representative structures of the GSL tilt angle. The blue and green lines indicate the vectors between the O1 atom of Glc1 moiety and the Sia3 and Sia6 nitrogen atoms, respectively.

Supporting Figures

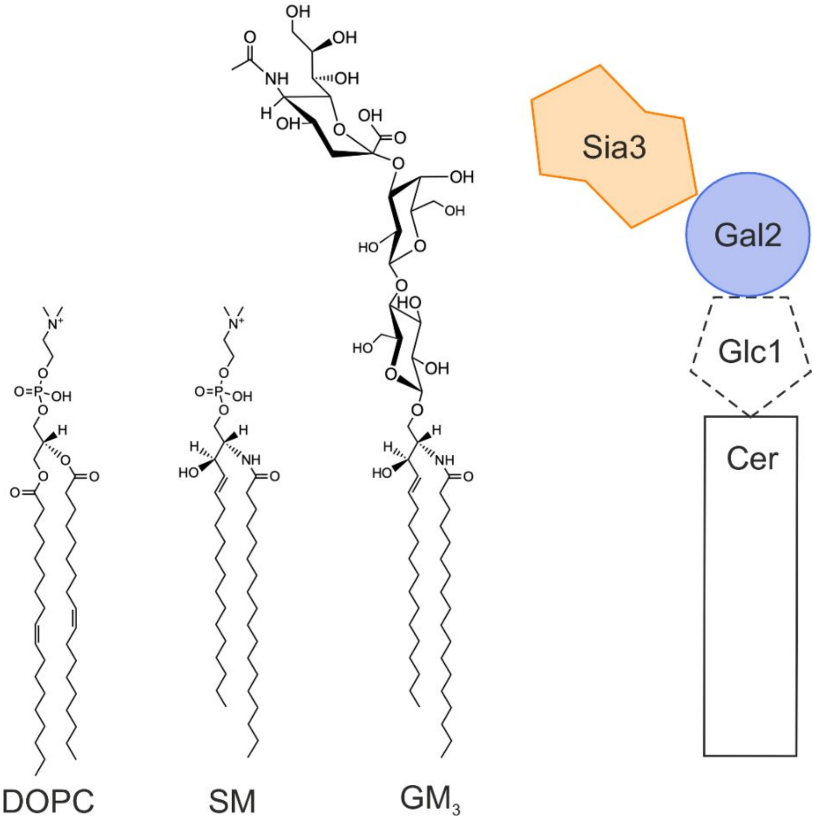

Figure SI5: Cartoon representation of the structure of dioleoyl phosphatidylcholine (DOPC), sphingomyelin (SM) and ganglioside GM_3 used in the manuscript. The building blocks of the glycan GM_3 headgroup are: glucose (Glc), galactose (Gal), sialic acid (Sia).

References

(1) Angelova, M. I.; Dimitrov, D. S. Liposome Electroformation. *Faraday Discuss.* **1986**, *81*, 303-+.
(2) Vinklárek, I. S.; Vel'as, L.; Riegerová, P.; Skála, K.; Mikhalyov, I.; Gretskaya, N.; Hof, M.; Šachl, R. Experimental Evidence of the Existence of Interleaflet Coupled Nanodomains: An Mc-Fret Study. *J. Phys. Chem. Lett.* **2019**, *10* (9), 2024-2030.
(3) Koukalová, A.; Amaro, M.; Aydogan, G.; Gröbner, G.; Williamson, P. T. F.; Mikhalyov, I.; Hof, M.; Šachl, R. Lipid Driven Nanodomains in Giant Lipid Vesicles Are Fluid and Disordered. *Sci Rep* **2017**, *7*, 12.
(4) Šachl, R.; Johansson, L. B. A.; Hof, M. Forster Resonance Energy Transfer (Fret) between Heterogeneously Distributed Probes: Application to Lipid Nanodomains and Pores. *Int. J. Mol. Sci.* **2012**, *13* (12), 16141-16156.
(5) Šachl, R.; Humpolíčková, J.; Štefl, M.; Johansson, L. B. A.; Hof, M. Limitations of Electronic Energy Transfer in the Determination of Lipid Nanodomain Sizes. *Biophys. J.* **2011**, *101* (11), L60-L62.
(6) Chmelová, B.; Davidović, D.; Šachl, R. Interleaflet Organization of Membrane Nanodomains: What Can(Not) Be Resolved by Fret? *Biophys. J.* **in press**. DOI: https://doi.org/10.1016/j.bpj.2022.11.014.
(7) Abraham, M. J.; Murtola, T.; Schulz, R.; Páll, S.; Smith, J. C.; Hess, B.; Lindahl, E. Gromacs: High Performance Molecular Simulations through Multi-Level Parallelism from Laptops to Supercomputers. *SoftwareX* **2015**, *1-2*, 19-25.
(8) Jo, S.; Kim, T.; Iyer, V. G.; Im, W. Charmm-Gui: A Web-Based Graphical User Interface for Charmm. *Journal of Computational Chemistry* **2008**, *29*, 1859-1865.

(9) Brooks, B. R.; Brooks, C. L.; Mackerell, A. D.; Nilsson, L.; Petrella, R. J.; Roux, B.; Won, Y.; Archontis, G.; Bartels, C.; Boresch, S.; et al. Charmm: The Biomolecular Simulation Program. *Journal of Computational Chemistry* **2009**, *30* (10), 1545-1614.
(10) Lee, J.; Cheng, X.; Swails, J. M.; Yeom, M. S.; Eastman, P. K.; Lemkul, J. A.; Wei, S.; Buckner, J.; Jeong, J. C.; Qi, Y. F.; et al. Charmm-Gui Input Generator for Namd, Gromacs, Amber, Openmm, and Charmm/Openmm Simulations Using the Charmm36 Additive Force Field. *J. Chem. Theory Comput.* **2016**, *12* (1), 405-413.
(11) Huang, J.; Rauscher, S.; Nawrocki, G.; Ran, T.; Feig, M.; de Groot, B. L.; Grubmuller, H.; MacKerell, A. D. Charmm36m: An Improved Force Field for Folded and Intrinsically Disordered Proteins. *Nat. Methods* **2017**, *14* (1), 71-73.
(12) Jorgensen, W. L.; Chandrasekhar, J.; Madura, J. D.; Impey, R. W.; Klein, M. L. Comparison of Simple Potential Functions for Simulating Liquid Water. *J. Chem. Phys.* **1983**, *79* (2), 926-935.
(13) Hess, B.; Bekker, H.; Berendsen, H. J. C.; Fraaije, J. Lincs: A Linear Constraint Solver for Molecular Simulations. *Journal of Computational Chemistry* **1997**, *18* (12), 1463-1472.
(14) Nose, S. A Unified Formulation of the Constant Temperature Molecular-Dynamics Methods. *J. Chem. Phys.* **1984**, *81* (1), 511-519.
(15) Hoover, W. G. Canonical Dynamics - Equilibrium Phase-Space Distributions. *Phys. Rev. A* **1985**, *31* (3), 1695-1697.
(16) Parrinello, M.; Rahman, A. Polymorphic Transitions in Single-Crystals - a New Molecular-Dynamics Method. *J. Appl. Phys.* **1981**, *52* (12), 7182-7190.
(17) Essmann, U.; Perera, L.; Berkowitz, M. L.; Darden, T.; Lee, H.; Pedersen, L. G. A Smooth Particle Mesh Ewald Method. *J. Chem. Phys.* **1995**, *103* (19), 8577-8593.
(18) Marushchak, D.; Gretskaya, N.; Mikhalyov, I.; Johansson, L. B. A. Self-Aggregation - an Intrinsic Property of G(M1) in Lipid Bilayers. *Mol. Membr. Biol.* **2007**, *24* (2), 102-112.
(19) Sarmento, M. J.; Owen, M. C.; Ricardo, J. C.; Chmelová, B.; Davidović, D.; Mikhalyov, I.; Gretskaya, N.; Hof, M.; Amaro, M.; Vácha, R.; et al. The Impact of the Glycan Headgroup on the Nanoscopic Segregation of Gangliosides. *Biophys. J.* **2021**, *120* (24), 5530-5543.
