# Supplementary material for: Which Moiety Drives Gangliosides to Form Nanodomains?
Source: J Phys Chem Lett. 2023 Jun 16;14(25):5791–7. doi: 10.1021/acs.jpclett.3c00761 (PMC10316399; doi:10.1021/acs.jpclett.3c00761)
Supplement: Supplementary file 2 — jz3c00761_si_002.pdf [file jz3c00761_si_002.pdf]

jz-2023-00761e.R1

Name: Peer Review Information for "Which Moiety Drives Gangliosides to Form Nanodomains?"

First Round of Reviewer Comments

Reviewer: 1

Comments to the Author

This is a really useful work studying the molecular details of ganglioside nanodomain formation. Authors showed, in a concise manner using MC-FRET, that Sialic acid residues are responsible for hydrogen bonding and nanodomain formation.

I think, this manuscript should be published in JPCL. My only suggestion is that the discussion can be improved to mention the sialic acid accessibility on the surface of membranes before and after nanodomain formation, which is critical for, e.g. host-pathogen interactions. This would give the work a bigger perspective and biological context.

Reviewer: 2

Comments to the Author

This paper investigates the influence of the Sia moiety on nanodomain formation by systematically investigating gangliosides with 0-4 Sia moieties in lipid bilayers containing DOPC/cholesterol/sphingomyelin/GSLs. Lipid compositions eliminating 1) sphingomyelin and 2) both cholesterol and sphingomyelin are also compared. The authors use a combination of Monte-Carlo FRET experiments and all-atom MD simulations to show that 1) the Sia residue is primarily responsible for the formation of nanodomains through formation of hydrogen bonds and 2) asialoGM$_1$ (containing no Sia residues) behaves more like sphingomyelin in nanodomain formation.

Gangliosides are an important component of many cellular processes at the membrane; however, the specific structural factors that influence their nanodomain formation have been difficult to elucidate. The combination of experimental (MC-FRET) and computational (MD simulations) techniques in this paper provide strong support for the Sia moiety as an influential factor in the formation of nanodomains, giving insight into the impact of headgroup structure on nanodomain formation. This is a significant advance as it is, to my knowledge, the first systematic study to directly show the influence of the Sia residue on nanodomain formation. However, I have two general concerns with the paper in its current form before it is suitable for publication in JPCL.

First, the MC-FRET technique relies on using 1 mol% (out of 5 mol% total GSL) BODIPY-conjugated GM$_1$ donor and acceptor FRET pairs to detect nanodomains in all samples. Since these GM$_1$ molecules contain Sia moieties, how does this affect the interpretation of the asialoGM$_1$ results? Could the BODIPY-GM$_1$

fluorescent probes influence the formation of nanodomains in asialoGM_1 samples? It is noted that asialoGM_1 forms no nanodomains in DOPC/GSL-only bilayers, but it is not clear how the nanodomains that are formed in the other two lipid conditions are influenced by the Sia-containing fluorescent probes. Control experiments either reducing the mol% of BODIPY-GM_1 donors and acceptors present or using fluorescent probes that lack the Sia residue to show any influence or lack thereof on nanodomain formation are needed. Providing the chemical structures of the lipid probes used in the SI would also be helpful to readers.

Second, I believe that the authors are too strong in their claim that they have "identif[ed] the minimal molecular requirements for ganglioside self-organization into nanodomains" (pg. 2, lines 49-50) and "The present results give the answer to an old question in membrane biophysics: 'It is the Sia moiety that drives gangliosides to form nanodomains.'" (pg. 8, lines 23-24). While this work provides strong evidence for the Sia moiety being a major influencer in nanodomain formation, it is not necessarily the only requirement for nanodomain formation in vivo. For example, in Arumugam, S., Schmieder, S., Pezeshkian, W. et al. Ceramide structure dictates glycosphingolipid nanodomain assembly and function. Nat Commun **12**, 3675 (2021). https://doi.org/10.1038/s41467-021-23961-9 the ceramide structure is found to influence GM_1 nanodomain formation. The authors should either tone down this claim or provide more support of their claim through discussion that considers literature showing other portions of the GSL structure (for example, ceramide structure) can be influential in nanodomain formation.

Specific comments:

1. Pg 5., lines 15-16 "whereas no nanodomains were observed for asialoGM1 ($\langle A \rangle$ close to 0), which is in line with the clustering behavior of SM" and Pg. 8, line 32 "For example, neither asialoGM1 nor SM form nanodomains in a homogeneous DOPC bilayer." - provide citations for this specific SM clustering behavior.

2. Pg. 3, lines 49-50: The specific lipid compositions of cholesterol and sphingomyelin were chosen because they are physiologically relevant. Citation(s) should be provided supporting the relevance of these numbers. In addition, it should be clarified in the text that the numbers chosen (65/25/10/5) are mole percentages.

3. Pg. 3, Line 45- I believe this should say "devoid of Sia" and not "devote of Sia".

4. Figure 1- The molecular structure of each building block should be shown in addition to the cartoon form. For example, the authors could include the full structure of GM_1, highlighting the portions that form each building block in the cartoon, and then show all of the GSLs (asialoGM_1, GM_1, GD_{1a}, GT_{1b}, GQ_{1B}) as cartoons as already depicted in the figure. Since data for GM_3 is also referenced in this paper, it would also be helpful to include its structure somewhere (either in Figure 1 or in the SI).

5. Table S1- Clarify in the table caption itself that the numbers in the table represent the number of lipids.

Author's Response to Peer Review Comments:

Dear Prof. Editor,

Thank you for careful evaluation of the submitted manuscript and also for providing a chance to improve the manuscript and clarify the ambiguities that have arisen. We greatly appreciated the suggestions of the reviewers, as we think that they helped to further improve the manuscript.

Our main effort was to revise the entire manuscript to remove all the ambiguities mentioned.

We would also like to point out that we have added a new author, Dr. Joana Ricardo, to the author list. Dr. Ricardo was initially involved in the project, but her input did not meet the criteria for authorship. However, at the revision stage, her contribution was more substantial, prompting us to include her in the list of authors. The filled authorship change form can be found in ACS paragon Plus and is divided into two pdf documents (lack of space).

Thank you for your attention to this manuscript,

Yours sincerely,

Radek Šachl and my colleagues

Responses to Editor's Comments to the Author (non-scientific changes)

1) Title: In both the main manuscript file and the Supporting Information, set the title in title case, with the first letter of each principal word capitalized.

The title has been modified accordingly both in the main manuscript and SI.

2) Main Text: Remove "Main Text" from page 2, line 11.

'Main text' has been removed.

*3) References: In both the main file and the supporting information, fix the style of all references to use JPCL formatting (check all references carefully). ***JPC Letters reference formatting requires that journal references should contain: () around numbers, author names, article title (titles entirely in title case or entirely in lower case), abbreviated journal title (italicized), year (bolded), volume (italicized), and pages (first-last). Book references should contain author names, book title (in the same pattern), publisher, city, and year. Websites must include date of access.*

We've gone through all the references again and they should now be in the correct format.

4) TOC Graphic: Please resize the TOC graphic per journal guidelines (2 in x 2 in) and move to the correct position (on the same page as the abstract).

TOC Graphic has been resized according to the instructions.

5) References: Upload any preprints in your reference list as Supporting Information for Review Only.

We have uploaded the requested file as *Supporting Information for Review Only and have added DOI number to the reference in question.*

6) Supporting Information Statement: A brief, nonsentence description of the actual contents of each supporting information file is required. This description should be labeled Supporting Information and should appear before the Acknowledgement and Reference sections. Examples of sufficient and insufficient descriptions are as follows:
**Examples of sufficient descriptions: "Supporting Information: 1H NMR spectra for all compounds" or "Additional experimental details, materials, and methods, including photographs of experimental setup".*
**Examples of insufficient descriptions: "Supporting Information: Figures S1-S3" or "Additional figures as mentioned in the text".*

A Supporting Information statement has been added to the appropriate place.

7) Supporting Information: Please number SI pages in the following format: "S1, S2…"

All pages have been numbered accordingly.

Responses to Reviewer's Comments to the Author

We would like to thank the reviewers for their comments and for the time taken to evaluate this manuscript. We are pleased with the overall positive evaluation of this paper. Our responses to all reviewer's comments are listed below.

Reviewer 1:

This is a really useful work studying the molecular details of ganglioside nanodomain formation. Authors showed, in a concise manner using MC-FRET, that Sialic acid residues are responsible for hydrogen bonding and nanodomain formation.

I think, this manuscript should be published in JPCL.

We very much appreciate this opinion.

My only suggestion is that the discussion can be improved to mention the sialic acid accessibility on the surface of membranes before and after nanodomain formation, which is critical for, e.g. host-pathogen interactions. This would give the work a bigger perspective and biological context.

We thank the reviewer for this comment. We have extended the discussion to give a few biological examples as follows:
'In the final part of this study, we set out to determine to which extent the above-described interactions of GSLs with the surroundings are influenced by the formation of the nanodomains. For example, ganglioside-mediated host-pathogen interactions (Wang et al, Front. Microbiol 2021; Cutillo et al, Cellular and Molecular Immunology 2020) that depend on the precise identification of ganglioside receptors by ganglioside-binding motifs depend on these interactions. More specifically, the dendritic cell protein Siglec-1 (Sialic Acid Binding Ig Like Lectin 1) recognizes gangliosides on the viral membrane of enveloped viruses like the human immunodeficiency virus (HIV)-1 or the Ebola virus (Peres-Zsolt et al, Viruses 2019) assisting in the propagation of viral infection and antiviral immune response. Another well-known example is the interaction of the cholera toxin protein with five GM_1 molecules when it approaches cellular plasma membranes. In this specific case, it has already been established that membrane composition and organization influence these interactions (Šachl, R. *et al.* Biochim. Biophys. Acta 1853, 850–7 (2015)).'

<u>Reviewer: 2</u>

This paper investigates the influence of the Sia moiety on nanodomain formation by systematically investigating gangliosides with 0-4 Sia moieties in lipid bilayers containing DOPC/cholesterol/sphingomyelin/GSLs. Lipid compositions eliminating 1) sphingomyelin and 2) both cholesterol and sphingomyelin are also compared. The authors use a combination of Monte-Carlo FRET experiments and all-atom MD simulations to show that 1) the Sia residue is primarily responsible for the formation of nanodomains through formation of hydrogen bonds and 2) asialoGM1 (containing no Sia residues) behaves more like sphingomyelin in nanodomain formation.

Gangliosides are an important component of many cellular processes at the membrane; however, the specific structural factors that influence their nanodomain formation have been difficult to elucidate. The combination of experimental (MC-FRET) and computational (MD simulations) techniques in this paper provide strong support for the Sia moiety as an influential factor in the formation of nanodomains, giving insight into the impact of headgroup structure on nanodomain formation. This is a significant advance as it is, to my knowledge, the first systematic study to directly show the influence of the Sia residue on nanodomain formation. However, I have two general concerns with the paper in its current form before it is suitable for publication in JPCL.

We very much appreciate this opinion. Regarding the reviewer's general comments, we have drafted the following responses.

First, the MC-FRET technique relies on using 1 mol% (out of 5 mol% total GSL) BODIPY-conjugated GM1 donor and acceptor FRET pairs to detect nanodomains in all samples. Since these GM1 molecules contain Sia moieties, how does this affect the interpretation of the asialoGM1 results? Could the BODIPY-GM1 fluorescent probes influence the formation of nanodomains in asialoGM1 samples? It is noted that asialoGM1 forms no nanodomains in DOPC/GSL-only bilayers, but it is not clear how the nanodomains that are formed in the other two lipid conditions are influenced by the Sia-containing fluorescent probes. Control experiments either reducing the mol% of BODIPY-GM1 donors and acceptors present or using fluorescent probes that lack the Sia residue to show any influence or lack thereof on nanodomain formation are needed.

In response to this comment we rely 1) on our already published results and 2) on additional experiments proposed by the reviewer:

1A) NO detectable impact of GM1 probes on sphingomyelin nanodomains: Most importantly, in Koukalová et al, Scientific Reports 2017, we have shown that the same donor-acceptor pair represented by Bodipy-FL-C5-GM1 (g-GM1, donor) and Bodipy-564/570-C5-GM1 (r-GM1, acceptor) used at the same probe to lipid concentration (1:200) does not affect clustering of sphingomyelin (a molecule closely related to asialo-GM1, and also devoid of any sialic acid; see **Table 1** adopted from Koukalová et al 2017). This reasoning was based on an experiment in which the size of sphingomyelin-induced nanodomains was determined using two completely different donor/acceptor pairs: Bodipy-FL-C5-GM1 (donor)/Bodipy-564/570-C5-GM1 and CF-PEG-DSPE (distearoyl phosphatidylethanolamine conjugated with PEG2000 labelled at the end of the pegylated chain by carboxyfluorescein)/Rh-PEF-DSPE (distearoyl phosphatidylethanolamine conjugated with PEG2000 labelled at the end of the pegylated chain by rhodamine). More specifically, for the first D/A pair, we obtained two global minima (R = 8 nm and Area = 37% and R = 12 nm and Area = 55%) and for the second D/A pair we obtained one minimum located at R = 9 nm and Area = 45 %. Because the determined nanodomain sizes and concentrations were similar for both D/A pairs, we concluded that the GM1 DA pair had negligible impact on the properties of sphingomyelin nanodomains.

Overall, we assume that if there is any perturbing effect of the GM1 DA pair on the properties of the nanodomains, this effect should be more pronounced for SM (containing no sugar in the headgroup and being structurally more distant from the molecular probe structure) than for asialo-GM1 (differing only in the absence of the sialic group and the presence of the Bodipy chromophore). Since no perturbing effect is evident in SM either, we assume that GM1 probes have a negligible effect on asialoGM1 nanodomains as well.

1B) NO detectable impact of GM1 probes on GM1 nanodomains: In Šachl et al BBA 2015, we investigated in detail a possible disturbing effect of GM1 probes on GM1 nanodomains. Specifically, we showed that GM1 probes do not spontaneously cluster bellow a total concentration of 1 mol% in homogeneous membranes. We deduced this from the fact that the obtained fluorescence decays were well fitted by the so-called Bauman -Fayer model considering a homogeneous distribution of donors and acceptors in the membrane (Baumann, J. *et al. J. Chem.*

Phys. 85, 4087–4107 (1986)). In the context of our new work, this behavior can be understood better because the fluorophores attached to the sialic group most likely suppress the ability of this group to form hydrogen bonds that we showed significantly stabilize ganglioside nanodomains.
In the same work, we demonstrated that GM1 nanodomains are formed at the same concentrations of unlabeled GM1 even after replacing Bodipy-FL-GM1 donors with a different dye, B7PC (1-palmitoyl-2-[(Me4-BODIPY-8)-acyl]-sn-glycero-3-phosphocholine with acyl containing 7 carbons).

Thus, since the GM1 probes do not show any demonstrable negative effect on both SM and GM1 nanodomains, we do not expect any interference even in the case of asialo-GM1 that is closely related to both sphingolipids.

DOPC (mol%)	SM (mol%)	Chol (mol%)	Domain radius (nm)	Domain area (%)	FRET pair	K_D(D)**	K_D(A)**	E_{rel}***
100, 75, 70	0	0, 25, 30	Homogeneous distrib.		g-GM_1/r-GM_1	-----*	-----*	1.00
95, 92	5, 8	0	Homogeneous distrib.		g-GM_1/r-GM_1	-----*	-----*	1.00
90, 88, 85	10, 12, 15	0	8 ± 1	37 ± 10	g-GM_1/r-GM_1	≈10	≈10	1.03
			12 ± 3	55 ± 10				
95, 92, 90, 88	5, 8, 10, 12	0	Homogeneous distrib.		CF-PEG-DSPE/Rh-PEG-DSPE	≈1	≈1	1.00
70, 67, 65	5, 8, 10	25	9 ± 1	45 ± 5	g-GM_1/r-GM_1	≥20	≥20	1.12
63	12	25	Homogeneous distrib.		g-GM_1/r-GM_1	-----*	-----*	1.00
70, 67	5, 8	25	Homogeneous distrib.		CF-PEG-DSPE/Rh-PEG-DSPE	≈1	≈1	1.00
65, 63	10, 12	25	8 ± 1	55 ± 5	CF-PEG-DSPE/Rh-PEG-DSPE	≈5	≈5	1.10
60	10	30	9 ± 1	45 ± 5	g-GM_1/r-GM_1	≥20	≥20	1.12

Table 1. The average radius and fractional bilayer area of the nanodomains, distribution constants K_D and relative FRET efficiencies E_{rel} (for definition see Materials and Method section) for two different donor-acceptor pairs in DOPC/SM and DOPC/Chol/SM mixtures. All lipid mixtures that are given in the same row provided overlapping fluorescence decays. For this reason, the same values are determined for these parameters in the mentioned bilayers. The output parameters were determined by MC-FRET. The total amount of D/A molecules was 1 mol% at max. *no nanodomains detected at the given bilayer compositions; **as determined by MC-FRET; ***the estimated error in Erel was below 1%.

2) NO detectable impact of GM1 probes on asialo-GM1 nanodomains: In the final part of this response, we have followed the advice of the reviewer and attempted to verify the aforementioned reasoning supporting the inert nature of GM1 probes for the case of asialo-GM1 nanodomains. For comparison, we performed the given experiments not only for asialo-GM1 but also for GM1 nanodomains and for simplicity we focused only on the DOPC/Chol binary mixtures. In the experiment, we reduced the donor concentration by a factor of 2 and 4, while the acceptor concentration could not be significantly manipulated in order to guarantee efficient excitation energy transfer, on which the sensitivity of the method depends significantly.

In this work, the nanodomain size and concentration was determined by the analysis of time-resolved fluorescence decays of Bodipy-FL-C5-GM1 donors in the presence of Bodipy-564/570-C5-GM1 acceptors. Thus, any change in the size or concentration of ganglioside nanodomains inevitably leads to a change in the fluorescence decays.

Figure R1 displays the outcomes of the tests that were conducted. Importantly, the shape of fluorescence decays for both GM1 and asialo-GM1 nanodomains remains constant while reducing Bodipy-FL-C5-GM1 concentration. As the absence of any change in the shape of the fluorescence decays indicates a constant nanodomain size and concentration, the result of this experiment is in agreement with our assumption that Bodipy-FL-C5-GM1 and Bodipy-564/570-C5-GM1 fluorescent probes do not influence the analyzed features of ganglioside nanodomains.

In conclusion, both our already published results and additional experiments proposed by the reviewer indicate that the used fluorescent probes have no discernable impact on the ganglioside nanodomains.

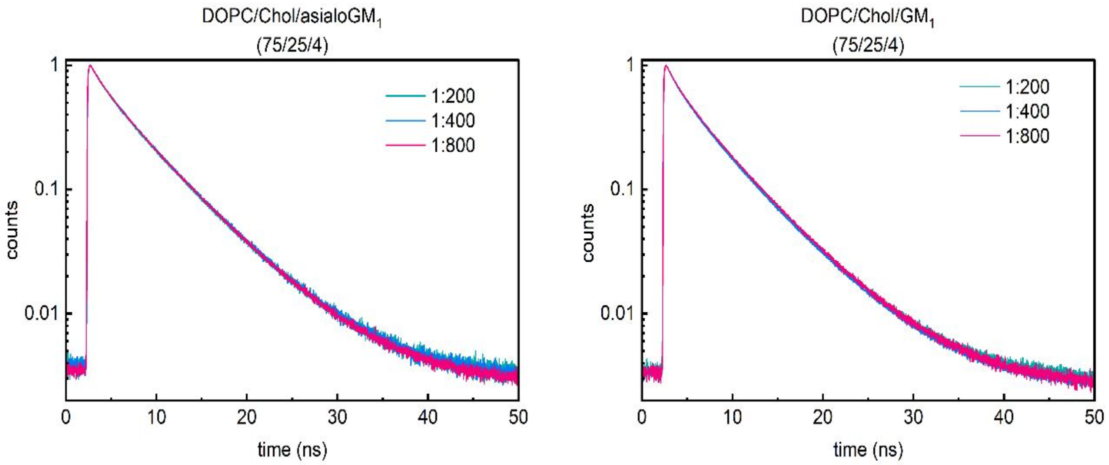

Figure R1: Time-resolved fluorescence decays of Bodipy-FL-C5-GM1 added at varying probe to lipid ratios in the presence of Bodipy-564/570-C5-GM1 acceptors (probe to lipid ratio 1:200). The shape of the decays reports on the size and surface concentration of asialo-GM1 (left) or GM1 (right) nanodomains in DOPC/Chol (75/25 mol%) membranes.

Providing the chemical structures of the lipid probes used in the SI would also be helpful to readers.

We agree with the reviewer that providing the chemical structures of ganglioside probes would be useful. Thus, the structures have been added as **Figure SI1** to SI.

Second, I believe that the authors are too strong in their claim that they have "identif[ed] the minimal molecular requirements for ganglioside self-organization into nanodomains" (pg. 2, lines 49-50) and "The present results give the answer to an old question in membrane biophysics: 'It is the Sia moiety that drives gangliosides to form nanodomains.'" (pg. 8, lines 23-24). While this work provides strong evidence for the Sia moiety being a major influencer in nanodomain formation, it is not necessarily the only requirement for nanodomain formation in vivo. For example, in Arumugam, S., Schmieder, S., Pezeshkian, W. et al. Ceramide structure dictates

*glycosphingolipid nanodomain assembly and function. Nat Commun **12**, 3675 (2021). https://doi.org/10.1038/s41467-021-23961-9 the ceramide structure is found to influence GM1 nanodomain formation. The authors should either tone down this claim or provide more support of their claim through discussion that considers literature showing other portions of the GSL structure (for example, ceramide structure) can be influential in nanodomain formation.*

We admit that the interactions in the ganglioside headgroup do not represent the only molecular 'force' that can drive the formation of ganglioside nanodomains. Nevertheless, as we showed in Sarmento et al, BiophysJ. 2021, the headgroup of GM1 is the major player in this process as it contributes by 65-67% to energetic stabilization of ganglioside nanodomains. We now also state this in the introduction on p3: '…*As expected, the bulky polysaccharide group has been proposed as the primary catalyst for the nanoscopic segregation of gangliosides. It contributes to the energetic stabilization of the nanodomains by 65-67%, with the hydrogen bonding network formed at the headgroup level being considered its main stabilizer…'*

As this letter focuses exclusively on the role of the bulky ganglioside headgroup in the segregation of gangliosides into nanodomains, we have followed reviewers recommendation a have chosen a more moderate tone in the above-mentioned sentences. Specifically,

1) We have replaced the sentence 'identif[ed] the minimal molecular requirements for ganglioside self-organization into nanodomains' (pg. 2, lines 49-50) by '…*identified the sialic acid moiety as a key group for self-organization of gangliosides into nanodomains, significantly advancing*…'
2) We have replaced the sentence 'The present results give the answer to an old question in membrane biophysics: 'It is the Sia moiety that drives gangliosides to form nanodomains.'" (pg. 8, lines 23-24) by '*Overall, these results show that the sialic group, which is an integral part of the bulky sugar headgroup of the ganglioside molecule, is crucially involved in the formation of ganglioside nanodomains***.**'
3) To highlight the complexity of the matter, we briefly mentioned in the introduction that also the hydrophobic ceramide part of gangliosides can induce reorganization of ganglioside nanodomains (Arumugam, S., Schmieder, S., Pezeshkian, W. et al. Ceramide structure dictates glycosphingolipid nanodomain assembly and function. Nat Commun **12**, 3675 (2021)): '…*The complexity of the matter is only underscored by the recent discovery that changes in the hydrophobic ceramide part of the molecule are also capable of inducing changes in the organization of gangliosides into nanodomains. It is thus not surprising that even today, the headgroups' molecular moiety driving the formation of ganglioside nanodomains has not yet been identified*…'

Specific comments:

1. Pg 5., lines 15-16 "whereas no nanodomains were observed for asialoGM1 ($\langle A \rangle$ close to 0), which is in line with the clustering behavior of SM" and Pg. 8, line 32 "For example, neither asialoGM1 nor SM form nanodomains in a homogeneous DOPC bilayer." - provide citations for this specific SM clustering behavior.

We apologize for omitting the following reference to an article in which we showed that sphingomyelin clusters in DOPC at concentrations greater than 8 mol% (Koukalová et al: Lipid Driven Nanodomains in Giant Lipid Vesicles are Fluid and Disordered, Scientific Reports 2017). We have added a reference to this article at both suggested locations of the paper.

2. Pg. 3, lines 49-50: The specific lipid compositions of cholesterol and sphingomyelin were chosen because they are physiologically relevant. Citation(s) should be provided supporting the relevance of these numbers. In addition, it should be clarified in the text that the numbers chosen (65/25/10/5) are mole percentages.

We thank the reviewer for picking this up. Although reports on the plasma membrane lipidome vary significantly depending on the cell type, tissue, technique and other experimental considerations, we chose these numbers as a way to keep cholesterol and sphingolipid levels within the ballpark of most of studies. To guide the reader to these various reports, and as suggested, we added several references to the manuscript. All compositions were also corrected to make clear they refer to mole percentages.

3. Pg. 3, Line 45- I believe this should say "devoid of Sia" and not "devote of Sia".

Thank you for having noticed this misprint. *"devote of Sia"* has been replaced by *"devoid of Sia".*

4. Figure 1- The molecular structure of each building block should be shown in addition to the cartoon form. For example, the authors could include the full structure of GM1, highlighting the portions that form each building block in the cartoon, and then show all of the GSLs (asialoGM1, GM1, GD1a, GT1b, GQ1B) as cartoons as already depicted in the figure. Since data for GM3 is also referenced in this paper, it would also be helpful to include its structure somewhere (either in Figure 1 or in the SI).

We agree with the reviewer that the molecular structure of each building block should be shown in **Figure 1**. We have therefore changed the image. An updated image can be found in the new version of this article. As for the structure of GM3, we have drawn it in **Figure SI5**.

5. Table S1- Clarify in the table caption itself that the numbers in the table represent the number of lipids.—

Thank you for this suggestion. We have revised the figure caption of **Table SI1** which now reads: '*Molecular composition of the simulated systems expressed in the number of lipid molecules present in the simulation box.*'

jz-2023-00761e.R1

Name: Peer Review Information for "Which Moiety Drives Gangliosides to Form Nanodomains?"

Second Round of Reviewer Comments

Reviewer: 2

Comments to the Author

The authors have sufficiently addressed my question about the possible influence of the lipid probes on the formation of nanodomains through both reference to previously published work and additional control experiments. My only suggestion is to directly mention this in the SI by 1) including a short statement that cites these references and 2) including Figure R1 as a figure in the SI so that readers are aware that this concern has been addressed.

The authors have also satisfactorily addressed my additional remarks, and the manuscript is improved. Therefore, I believe this paper is suitable for publication in JPCL subject to the minor addition to the SI as stated above.
